# Proteases—From Basic Structure to Function to Drug Design as Targeted Therapy

**DOI:** 10.3390/biology11111680

**Published:** 2022-11-21

**Authors:** Hang Fai Kwok, Brian Walker, Chris Shaw

**Affiliations:** 1 Institute of Translational Medicine, Faculty of Health Sciences, University of Macau, Avenida de Universidade, Taipa, Macau SAR 999078, China; 2 Department of Biomedical Sciences, Faculty of Health Sciences, University of Macau, Avenida de Universidade, Taipa, Macau SAR 999078, China; 3 School of Pharmacy, Queen’s University Belfast, Belfast BT9 7BL, UK

In the last two decades, proteases have become a primary and vital target in drug discovery. The U.S. FDA has approved more than 12 protease therapies in the previous 12 years, and many next-generation or completely new protease inhibitor drugs are under clinical development. Protease inhibition strategies comprise one of the fastest-expanding areas in the field of drug development and show considerable promise. In this Special Issue, we focused on the recent advances in the discovery and development of protease inhibitors, covering their synthesis, the design of new chemical entities acting as inhibitors of unique/particular types of proteases, and their mode of action.

Nuclear matrix metalloproteinases are emerging with distinct functions in several pathological conditions and physiological processes. The article by Frolova and her team provides an excellent review on the progress that has been made in this area of research and discusses the potential for their targeted inhibition for future therapeutic development [1].

Changes in matrix metalloprotease (MMP) expression levels are often considered surrogate markers for cancer progression, and reduced matrix metalloprotease levels upon treatment are regarded as proof of the anti-tumorigenic potential of the mediator under study. In the article by Slapak and his team, they investigate whether MMP expression drives pancreatic cancer progression and whether decreased MMP levels in experimental settings indicate treatment response. They conclude that it is not as convincing as expected, and that while individual MMPs may contribute to pancreatic cancer growth and metastasis, the current data on pancreatic ductal adenocarcinoma (PDAC) do not support the generalized notion that all MMPs drive PDAC progression [2].

Another review article by Kunnapuu and his team described how vascular endothelial growth factors (VEGFs) regulate the growth of blood and lymphatic vessels. Some VEGFs induce the growth of blood vessels, and others are responsible for the development of lymphatic vessels. Blocking VEGF-A is used today to treat several types of cancer as anti-angiogenic therapy. However, in other diseases, scientists aim to increase the activity of VEGFs. For example, VEGF-A could generate new blood vessels to protect from heart disease, and VEGF-C could develop new lymphatics to counteract lymphedema. Some of the latest clinical trials are currently testing the latter concept. Because VEGF-C and VEGF-D are produced as inactive precursors, Kunnapuu and his team propose that novel drugs could also target the enzymatic activation of VEGF-C and VEGF-D. However, the delicate balance between too much and too little vascular growth requires a detailed understanding of the activation of VEGFs before such concepts can be translated into efficacious and safe clinical therapies [3].

Two research papers regarding the new applications of serine and cysteine proteases as bioactive inhibitors have been discussed and described by Wang et al. and Bartošová-Sojková et al., respectively. Wang and her team developed two analogues of novel Bowman–Birk-type inhibitors named ranacyclin-NF1 (RNF1) and ranacyclin-NF3L (RNF3L) based on the features of related inhibitors from *Odorrana grahami* (ORB) and *Rana esculenta* (ranacyclin-T). In addition, they performed detailed structure-activity studies and demonstrated that residues outside the trypsin inhibitory loop (TIL) are related to the efficacy of trypsin inhibitory activity [4]. Another research study by Bartošová-Sojková and her team revealed that cysteine protease inhibitors from early emerging animal groups are highly diverse, with modifications in gene organization and protein architecture. The team set out hypotheses to describe the driving forces for the origins of this unique cystatin subtype and propose evolutionary scenarios elucidating the current existence of cystatins in the Metazoa, especially in their early emerging lineages. Their study identified molecules for which future functional studies could help to identify their roles in parasites and host–parasite interactions [5].
